# Identification of HLA-A*02-B*46 haplotype allele variant in Guangdong Han populations on the basis of PCR-SBT

**DOI:** 10.1186/1756-0500-2-55

**Published:** 2009-04-07

**Authors:** Fu Xiong, Lulu Xiao, Min Luo, Fei Huang

**Affiliations:** 1 The Department of Tissue Typing Center, Nanfang Hospital, Southern Medical University, Guangzhou 510515, Guangdong, PR China; 2 The Department of Medical Genetics, School of Basic Medical Sciences, Southern Medical University, Southern Medical University, Guangzhou 510515, Guangdong, PR China

## Abstract

**Background:**

The HLA-A*02-B*46 haplotype is one of most frequent haplotypes among Guangdong Han populations. To explore the characteristics of the HLA-A*02-B*46 haplotype in Guangdong Han populations, the genetic polymorphism of HLA-A*02-B*46 haplotype was analysed by PCR-SBT in our study.

**Findings:**

Among 88 samples with the homozygotes for HLA-A*02-B*46 in the low resolution, 4 different alleles for A*02 (A*0201, A*0203, A*0206, A*0207) and 1 allele for B*46 (B*4601) were identified by PCR-SBT. Among them, the A*0207 allele was the predominant allele. Inversely, among the samples with HLA-A*2-B*46(-), six alleles were detected for A*02 (A*020101, A*0203, A*0205, A*0206 and A*0207), and the A*0201 allele was predominant. On the other hand, the HLA-A*02-B*46 haplotype presented moderate heterozygosis (32.95%). In addition, the linkage with DRB1 was analysed in HLA-A*2-B*46 haplotype, and there existed 10 alleles with DRB1. With the low resolution for DRB1, the other 10 DRB1 alleles all linked with the HLA-A*02-B*46 haplotype except for DRB1*01, DRB1*10, and DRB1*17. Moreover, we found eight alleles of DRB1 in the HLA-A*0207-B*4601 haplotype.

**Conclusion:**

The polymorphism distribution of the A*02 allele between the HLA-A*02-B*46 and HLA-A*02-B*46(-) haplotypes among the Guangdong Han populations provides useful information for research on unrelated hematopoietic stem cell transplantation (UHSCT), anthropology, and disease association for populations with the HLA-A*02-B*46 haplotype.

## Background

HLA-A*02 is the most common allele in humans [1,2], and is the most heterogeneous HLA-A specificity, with 97 subtypes [3]. HLA-B*46 is the most common allele in East Asian populations and occurs at varying frequencies in different populations, including 14% among the Thais, 13.2% among the Vietnamese, 4.4% among the Japanese, and 10% among the Chinese [4]. The HLA-A*02-B*46 haplotype is one of most common haplotypes in Chinese populations, especially the Guangdong Han population. HLA-A*02-B*46 presents significant linkage disequilibrium, which shows its genetic dominance. Moreover, it is shown that the HLA-A*02-B*46 allele is positively related to certain diseases, such as nasopharyngeal carcinoma, which has a high incidence in Guangdong province [5,6].

The intent of this study was to explore the polymorphism of the HLA-A*02-B*46 haplotype homozygote and its distribution characteristics in the Guangdong Han population, and also to establish a bone marrow bank with HLA-A*02-B*46 haplotype to improve the effect of bone marrow transplantation and decrease the occurrence of GVHD. Therefore, we have analysed the genetic polymorphism of HLA-A*02-B*46 in Guangdong Han population by polymerase chain reaction-sequence-based typing (PCR-SBT) and report our findings in this paper.

## Methods

### Subjects

For all subjects, genomic DNA was isolated from white cells in 5 ml peripheral blood using the DNA Extraction Kit (Qiagen) according to the manufacturer's specifications. Primary genotyping was carried out by PCR-RSSO (Dynal Biotech Ltd, Invitrogen) on 8191 blood samples from unrelated bone marrow donors from the Guangdong Han population. Of the 8191 donors, 88 [mean age (35.5 ± 7.8) years, range 18~55 years] that were typed as HLA-A*02-B*46 positive were used in the present investigation. They were all ethnic Han, and their families have lived in Guangdong Province for at least three generations. As a control, 150 samples with A*02 and without the HLA-B*46 allele were selected in research. These selected samples were homozygotes for HLA-A*02-B*46 in the low resolution (PCR-RSSO). The local ethics committee reviewed the study and informed consent was obtained from all the participants.

### HLA-A*02-B*46 genotyping

HLA typing was performed by PCR-SBT analysis as previously described after amplifying the second exon of HLA-A, the third exon of HLA-B, and the second exon of HLA-DRB1 [7]. High resolution typing of the HLA-A*02, B*46, and DRB1 alleles was performed by PCR-SBT using the ABI 3100 with the software Matchtool. The primers used for PCR-amplification and sequencing of HLA-A, B*46, DRB1 were listed in table 1 and 2, respectively.

**Table 1:** Sequence specific oligonucleotide used for PCR-amplification of HLA-A, B*46, DRB1

Gene Names	Primer Names	Nucleotide sequence(5'-3')	Tm°	Position
HLA-A	Aintron1F	5'-CTC TG(C/T) GGG GAG AAG CAA-3'	58	4864–4881^a^
	Aex5mod R	5'-CCA GCA A(G/T)G ATG CCC ACG AT-3'	62	6735–6754^a^
HLA-B	BXI F	5'-GGG AGG AGC GAG GGG ACC (C/G)CA G-3'	74	5624–5645^b^
	BINT R	5'-GGA GGC CAT CCC CGG CGA CCT AT-3'	78	4703–4725^b^
HLA-DRB1*01	DRB01F	5'-TCC CAG TGC CCG CTC CCT-3'	62	-,*
	DRB01 R	5'-ACA CAC TCA GAT TCT CCG CTT-3'	62	-,*
HLA-DRB1*15,16	DRB02 F	5'-GGT GGG TGC TGT TGA AGG T-3'	60	-,*
	DRB02 R	5'-ACA CAC ACA CTC AGA TTC CCA-3'	62	-,*
HLA-DRB1*03 (exclude0317), 1402, 1406, 1413	DRB03 F	5'-AGC ACT AAG GAA GGG TTC AG-3'	60	-,*
	DRB03 R	5'-ACA CAC ACA CTC AGA TTC CCA-3'	62	-,*
HLA-DRB1*04	DRB04 F	5'-CCT GGG ATC AGA GGT AGA TTTT-3'	62	-,*
	DRB04 R	5'-ACA CAC ACA CTC AGA TTC TCC-3'	62	-,*
HLA-DRB1*07	DRB07 F	5'-CGG CGT CGC TGT CAG TGT T-3'	62	-,*
	DRB07 R	5'-TCA GAT TCC CAG CTC GGA GA-3'	62	-,*
HLA-DRB1*08	DRB08 F	5'-AGC GCA GGC CAG GCT CAA A T-3'	62	-,*
	DRB08 R	5'-ACA CAC ACA CTC AGA TTC CCA-3'	62	-,*
HLA-DRB1*09	DRB09 F	5'-CAG TTA AGG TTC CAG TGC CA-3'	60	-,*
	DRB09 R	5'-ACA CAC ACA CTC AGA TTC CCA-3'	62	-,*
HLA-DRB1*10	DRB10 F	5'-GGC GTT GCG GGT CGG CG-3'	62	-,*
	DRB10 R	5'-ACA CAC AGA GTC AGA TTC CCA-3'	62	-,*
HLA-DRB1*03(exclude0317), 11, 13, 14	DRB11.1 F	5'-TGG TGG GCG TTG GGG CG-3'	60	-,*
	DRB11.1 R	5'-ACA CAC ACA CTC AGA TTC CCA-3'	62	-,*
HLA-DRB1*11, 13(exclude1313), 14	DRB11.2 F	5'-AGC ACT AAG GAA GGG TTC AC-3'	60	-,*
	DRB11.2 R	5'-TGT CAC CTC CCC ACA GAG T-3'	60	-,*
HLA-DRB1*03, 11,13(exclude1313&0317)	DRB11.3 F	5'-GTT TTC CCG CCT GGT CCC C-3'	64	-,*
	DRB11.3 R	5'-TGT CAC CTC CCC ACA GAG T-3'	60	-,*
HLA-DRB1*12	DRB12 F	5'-AAC AGG CTG GAG GTA CGG AC-3'	64	-,*
	DRB12 R	5'-ACA CAC ACA CTC AGA TTC CCA-3'	62	-,*
HLA-DRB1*1301,1302	DRB13.1 F	5'-GTG GGC GTT GCG GCG GC-3'	66	-,*
	DRB13.1 R	5'-ACA CAC ACA CTC AGA TTC CCA-3'	62	-,*
HLA-DRB1*14(exclude1402, 1406, 1413)	DRB14 F	5'-GTT TTC CCG CCT GGA CCC T-3'	62	-,*
	DRB14 R	5'-ACA CAC ACA CTC AGA TTC CCA-3'	62	-,*
HLA-DRB1*07	DRB07 F	5'-CGG CGT CGC TGT CAG TGT T-3'	62	-,*
	DRB07 R	5'-TCA GAT TCC CAG CTC GGA GA-3'	62	-,*
HLA-DRB1*08	DRB08 F	5'-AGC GCA GGC CAG GCT CAA A T-3'	62	-,*
	DRB08 R	5'-ACA CAC ACA CTC AGA TTC CCA-3'	62	-,*
HLA-DRB1*09	DRB09 F	5'-CAG TTA AGG TTC CAG TGC CA-3'	60	-,*
	DRB09 R	5'-ACA CAC ACA CTC AGA TTC CCA-3'	62	-,*
HLA-DRB1*10	DRB10 F	5'-GGC GTT GCG GGT CGG CG-3'	62	-,*
	DRB10 R	5'-ACA CAC AGA GTC AGA TTC CCA-3'	62	-,*

^a^, Numbering of the HLA-A sequence is from the GeneBank accession NT_007952

^b^, Numbering of the HLA-B sequence is from the GeneBank accession NT_007592

*, Numbering of the HLA-DRB1 sequence is from the reference 7 and

**Table 2:** Sequence specific oligonucleotide used for sequencing of HLA-A*02, B*46 and DRB

Gene Names	Primer Names	Nucleotide sequence(5'-3')	Tm°	Position
HLA-A Exon 2	A5.9 2F	5'-TCG GGC (A/G)GG TCT CAG CC-3'	60	4934–4950^a^
	AEx2 2R	5'-CAC TCA CCG GCC TCG CTC TGG-3'	68	5222–5242^a^
HLA-A Exon 3	A5.10 3F	5'-GGG CTC GGG GGA C(C/T)G GG-3'	64	5438–5454^a^
	A3.4 3R	5'-GAG GCG CCC CGT GGC-3'	58	5762–5776^a^
HLA-B Exon 2	BEX 2F	5'-GGG CGC AGG ACC (T/C)G(A/G) GGA-3'	58	5602–5619^b^
	BEX 2R	5'-GGT CAC TCA CCG (G/T)CC TCG-3'	58	5272–5289^b^
HLA-B Exon 3	BEX 3F	5'-GGG GCC AGG GTC TCA CA-3'	58	5028–5044^b^
	BEX 3R	5'-CCC ACT GCC CCT GGT ACC-3'	62	4744–4761^b^
HLA-DRB1 Exon 2	21M13F	5'-TGT AAA ACG ACG GCC AGT-3'	54	-,*
	M13R	5'-CAG GAA AGA GCT ATG ACC-3'	54	-,*

^a^, Numbering of the HLA-A sequence is from the GeneBank accession NT_007952

^b^, Numbering of the HLA-B sequence is from the GeneBank accession NT_007592

*, Numbering of the HLA-DRB1 sequence is from the reference 7 and

### Statistical analysis

Relative frequencies of the HLA-A*2-B*46 allele were calculated by direct counting. χ^2 ^analysis was used to assess statistically significant differences between the HLA-A*02-B*46 and HLA-A*02-B*46(-) haplotype, Pc is obtained by P multiplying the number of alleles at each locus. Statistical analyses were conducted with an SPSS software program.

## Results

### Allele distribution of the A*02 and B*46 genes in the HLA-A*02-B*46 haplotype

In the 88 samples, 176 A*02-B*46 haplotypes were detected. Only four A*2 alleles including A*0201, A*0203, A*0206, A*0207 were detected in 176 HLA-A*02-B*46 haplotypes. Of the four A*2 alleles, A*0207 allele was the predominant allele with a frequency of 77.3%, followed by A*0201, A*0203, and A*0206 with respective frequencies of 14.2%, 4.0% and 4.5% (Table 3). In the control group, six A*02 alleles were detected, including A*0201, A*0203, A*0205, A*0206, A*0207 and A*0210; the A*0201 allele was predominant with a frequency of 55.3% (Table 3). The A*0205 and A*0210 alleles were not detected in the HLA-A*02-B*46 haplotype. Additionally, only one B*46 alleles with B*4601 was observed in 176 A*02-B*46 haplotypes. There existed significant difference with the frequencies of A*2 subtypes between HLA-A*02-B*46 haplotype and HLA-A*02-B*46(-) haplotype.

**Table 3:** Compared with the polymorphism of alleles between HLA-A*02-B*46 haplotype and HLA-A*02-B*46(-) haplotype

A*02 Allele	HLA-A*02-B*46		HLA-A*02-B*46(-)		Pc
		
	Number	RF(%)	Number	RF(%)	
A*0201	22	12.5	83	55.3*	Pc < 0.05
A*0203	9	5.1	9	6	Pc > 0.05
A*0205	0	0	5	3.3*	Pc < 0.05
A*0206	12	6.8	18	12*	Pc < 0.05
A*0207	133	75.6	32	21.3*	Pc < 0.05
A*0210	0	0	3	2*	Pc < 0.05
Total	176	100	150	100	Pc < 0.05

* Pc < 0.05, significantly different compared with HLA-A*02-B*46.

### Heterozygosis of A*02-B*46 haplotype in the Guangdong Han population

HLA-A*02-B*46 haplotype presents homozygosis at low resolution. However, at high resolution, HLA-A*02-B*46 haplotype possess moderate heterozygosis and the ratio is 32.95%. Although A*02 presents a polymorphism in HLA-A*02-B*46 haplotypes, the ratio of heterozygosis with A*02 is low. The major homozygous alleles in the HLA-A*02-B*46 haplotypes of the Guangdong Han population are HLA-A*0207-B*4601 and A*0207-B*4601, and the major heterozygotes are HLA-A*0201-B*4601 and HLA-A*0207-B*4601 (Table 4).

**Table 4:** The heterozygosis of HLA-A*02-B*46 haplotype

HLA-A*02-B*46 haplotype	A*0201	A*0203	A*0206	A*0207
A*0201	2	2	2	14
A*0203		1	2	3
A*0206			1	6
A*0207				55

### The diversity of the DRB1 allele linked to the HLA-A*02-B*46 haplotype

From 55 homozygotes for HLA-A*02-B*46 haplotype, the polymorphism of the DRB1gene was analyzed. We found 10 DRB1 alleles, including DRB1*04, DRB1*07, DRB1*08, DRB1*09, DRB1*11, DRB1*12, DRB1*13, DRB1*14, DRB1*15, DRB1*15 and DRB1*16 (Table 5). Among the 10 DRB1 alleles, the DRB1*09 is the most common allele in the Guangdong Han population. A polymorphism of the DBR1 gene was detected in the HLA-A*0207-B*4601 haplotype that existed as 8 DRB1 alleles, including DRB1*04, DRB1*08, DRB1*09, DRB1*11, DRB1*12, DRB1*14, DRB1*15, DRB1*16 (Table 5). Among the 8 DRB1 alleles, the DRB1*09 is also the most common allele in the HLA-A*0207-B*4601 haplotype.

**Table 5:** The polymorphism of DRB1 allele linked by HLA-A*02-B*46 haplotype and HLA-A*0207-B*4601 haplotype

	HLA-A*02-B*46-DRB1		HLA-A*0207-B*4601-DRB1	
	
DRB1Allele	Number	RF(%)	Number	RF(%)
DRB1*04	11	6.25	2	1.82
DRB1*07	4	2.27	0	0
DRB1*08	12	6.82	14	12.73
DRB1*09	114	64.78	73	66.36
DRB1*11	7	3.98	5	4.55
DRB1*12	5	2.84	5	4.55
DRB1*13	4	2.27	0	0
DRB1*14	10	5.68	4	3.63
DRB1*15	6	3.41	4	3.63
DRB1*16	3	1.7	3	2.73
Total	176	100	110	100

## Discussion

The HLA-A*02-B*46 haplotype is one of most frequent haplotypes found in Southern Chinese Han populations, especially in the Guangdong Han population. The HLA-A*02-B*46 haplotype has been reported to possibly be related to the genesis of nasopharyngeal carcinoma, the most common cancer in Guangdong province [5,6]. By means of linkage analysis of the HLA-A*02-B*46 haplotype, it may be possible to detect NPC genetic susceptibility cases in NPC high risk families. In our study, the HLA-A*02, HLA-B*46, HLA-DRB1 alleles and HLA-A*02-B*46 haplotypes in the Guangdong Han population were analysed by high-resolution SBT to study their genetic background. This approach was more efficient than traditional genetic methods, which were based on sequence-specific oligonucleotide probe (SSOP) and sequence-specific primers (SSP).

The HLA-A*02-B*46 haplotype showed high genetic diversity in the Guangdong Han population because of the polymorphism of A*02, although we found only one B*46 allele with B*4601 in HLA-A*02-B*46 haplotypes. HLA-A*02 alleles can be classified into two subgroups, including A*0201 and A*0205, by the diversity of the DNA sequence. Almost all populations have A*0201; however, A*0203, A*0206, A*0207 and A*0210 have only been found in Oriental populations, while A*0205, A*0208 and A*0209 are known to exist only in white populations. Furthermore, A*0202 and A*0214 have been found to exist only in black populations, while A*0211 are usually found only in Orientals and among South American Indians [3,8-10]. In our research, only four A*02 alleles were detected in the HLA-A*02-B*46 haplotype and the A*0205 and A*0210 alleles were not detected. It may be that A*0205 and A*0210 alleles do not exist in the HLA-A*02-B*46 haplotype. On the other hand, we found that the A*0207 allele was the predominant allele (77.3%) in the HLA-A*02-B*46 haplotype. It has been shown that HLA-A*0207-B*4601 is the most common subtype in the HLA-A*02-B*46 haplotype within the Guangdong Han population. However, when compared with the HLA-A*02-B*46(-) haplotype, the polymorphism of the A*02 allele shows a conspicuous diversity in the HLA-A*02-B*46 haplotype. The A*0201 allele was found to be the predominant allele (55.3%) in the HLA-A*02-B*46(-) haplotype. Conversely, the A*0207 allele had a measured frequency of only 21.3%. Therefore, the polymorphism distribution of A*02 allele between the HLA-A*02-B*46 and HLA-A*02-B*46(-) haplotypes is significantly different within the ethnic Han population. Most studies have shown that A*0201 is the prevalent gene in Chinese Han populations and, therefore, is not useful as a genetic marker. However, some investigations have found that at higher resolution, A*0201 does present diversity between North and South Chinese Han populations which could be regarded as a genetic marker [11,12]. In our study, the frequency of A*0201 presents diversity between different haplotypes, so further investigations of the subtypes are important to determine the frequency of A*0201.

High-resolution SBT was applied to analyse the alleles of HLA-A*02-B*46 in our study; this technique has shown that the HLA-A*02-B*46 presents heterozygosis to some extent because of the diversity associated with the A*02 allele. However, the frequency of heterozygosis was lower than the HLA-A*02 haplotype, which has been reported to show 85% heterozygosis [11]. The cause may be the high proportion of A*0207-B*4601. The low heterozygosity of HLA-A*02-B*46 makes it easy to look for unrelated donors with HSCT to transplant into patients with the HLA-A*02-B*46 haplotype.

We have found 10 DRB1 alleles in the HLA-A*02-B*46 haplotype. With the low resolution for DRB1, the other DRB1 alleles all exhibited linked behaviour and linked with the HLA-A*02-B*46 haplotype except for DRB*01, DRB*10, and DRB*17. However, there were only 8 DRB1 alleles in the HLA-A*0207-B*4601 haplotypes, as DRB*07 and DRB*13 were not detected in the HLA-A*0207-B*4601 haplotypes in our study. DRB1*08 has been reported to be a common extended HLA-A*02-B*46-DRB1 haplotype among the Japanese and Koreans (24.40% in frequency) [12]; however, DRB1*09 was the most common allele in the HLA-A*02-B*46 haplotype in South-East Asia [13]. Our results indicate that the DRB1*09 is also the most common allele in the HLA-A*02-B*46-DRB1 haplotype within the Guangdong Han population, with a frequency of 66.36%

In conclusion, the polymorphism and distribution of HLA-A*02-B*46 haplotypes in the Guangdong Han population shares some genetic characteristics with other populations in other parts of China or other countries. These data provide useful information for unrelated hematopoietic stem cell transplantation (UHSCT), anthropology, and disease association studies.

## Abbreviations

HLA: Human Leukocyte Antigen; RF: relative frequency.

## Competing interests

The authors declare that they have no competing interests.

## Authors' contributions

FX and FH collectively conceived the study, designed the experiment; FX wrote the manuscript; FH prepared the tables for the manuscript; FH and ML carried out PCR and HLA-A*02-B*46 genotyping. LX contributed to the conception and design of the experiment and were also involved in editing the manuscript. All of the authors have read and approved the final manuscript.
